# IDH-mutant astrocytomas with primitive neuronal component have a distinct methylation profile and a higher risk of leptomeningeal spread

**DOI:** 10.1007/s00401-025-02849-8

**Published:** 2025-02-03

**Authors:** Felix Hinz, Dennis Friedel, Andrey Korshunov, Franziska M. Ippen, Henri Bogumil, Rouzbeh Banan, Sebastian Brandner, Martin Hasselblatt, Henning B. Boldt, Vaidas Dirse, Hildegard Dohmen, Eleonora Aronica, Michael Brodhun, Marike L. D. Broekman, David Capper, Asan Cherkezov, Maximilian Y. Deng, Vera van Dis, Jörg Felsberg, Stephan Frank, Pim J. French, Rüdiger Gerlach, Kirsten Göbel, Eric Goold, Jürgen Hench, Sven Kantelhardt, Patricia Kohlhof-Meinecke, Sandro Krieg, Christian Mawrin, Gillian Morrison, Angelika Mühlebner, Koray Ozduman, Stefan M. Pfister, Pietro Luigi Poliani, Marco Prinz, Guido Reifenberger, Markus J. Riemenschneider, Roman Sankowski, Daniel Schrimpf, Martin Sill, Matija Snuderl, Robert M. Verdijk, Mathew R. Voisin, Pieter Wesseling, Wolfgang Wick, David E. Reuss, Andreas von Deimling, Felix Sahm, Sybren L. N. Maas, Abigail K. Suwala

**Affiliations:** 1 Department of Neuropathology, Institute of Pathology, University Hospital Heidelberg, Im Neuenheimer Feld 224, 69120 Heidelberg, Germany; 2 Clinical Cooperation Unit Neuropathology, German Consortium for Translational Cancer Research (DKTK), German Cancer Research Center (DKFZ), Heidelberg, Germany; 3 Hopp Children’s Cancer Center Heidelberg (KiTZ), Heidelberg, Germany; 4 Department of Neurology, Heidelberg University Hospital, Heidelberg, Germany; 5 National Center for Tumor Diseases (NCT), NCT Heidelberg, a Partnership Between DKFZ and Heidelberg University Hospital, Heidelberg, Germany; 6 Division of Neuropathology, National Hospital for Neurology and Neurosurgery, University College London Hospitals NHS Foundation Trust, Queen Square, London, UK; 7 Department of Neurodegenerative Disease, UCL Queen Square Institute of Neurology, Queen Square, London, UK; 8 Institute of Neuropathology, University Hospital Münster, Münster, Germany; 9 Department of Pathology, Odense University Hospital, Odense, Denmark; 10 Department of Clinical Research, University of Southern Denmark, Odense, Denmark; 11 Hematology, Oncology and Transfusion Medicine Center, Vilnius University Hospital Santaros Klinikos, Vilnius, Lithuania; 12 Institute of Neuropathology, Justus-Liebig University Giessen, Giessen, Germany; 13 Department of Pathology, Amsterdam University Medical Centers (UMC), University of Amsterdam, Amsterdam Neuroscience, Amsterdam, The Netherlands; 14 Department of Neurosurgery, Helios Clinic Erfurt, Erfurt, Health and Medical University, Erfurt, Germany; 15 Department of Pathology and Neuropathology, Helios Clinic Erfurt, Erfurt, Health and Medical University, Erfurt, Germany; 16 Department of Neurosurgery, Haaglanden Medical Center and Leiden University Medical Center, Leiden, The Netherlands; 17 Department of Neuropathology, Charité-Universitätsmedizin Berlin, Corporate Member of Freie Universität Berlin and Humboldt-Universität zu Berlin, Berlin, Germany; 18 German Cancer Consortium (DKTK), Partner Site Berlin, German Cancer Research Center (DKFZ), Heidelberg, Germany; 19 Translational Pediatric Radiation Oncology, Hopp Children’s Cancer Center (KITZ), Heidelberg, Germany; 20 Department of Radiation Oncology, Heidelberg University Hospital, Heidelberg University, Heidelberg, Germany; 21 Heidelberg Institute for Radiation Oncology (HIRO) and National Center for Radiation Research in Oncology (NCRO), Heidelberg, Germany; 22 Department of Radiation Oncology, Heidelberg Ion-Beam Therapy Center (HIT), Heidelberg University Hospital, Heidelberg University, Heidelberg, Germany; 23 Department of Pathology, Leiden University Medical Center, Leiden, The Netherlands; 24 Department of Pathology, Erasmus Medical Center, Rotterdam, The Netherlands; 25 Institute of Neuropathology, Medical Faculty, and University Hospital Düsseldorf, Heinrich Heine University, and German Cancer Consortium (DKTK), Partner Site Essen/Düsseldorf, Düsseldorf, Germany; 26 Institute of Pathology, Division of Neuropathology, University Hospital Basel, University of Basel, Basel, Switzerland; 27 Department of Neurology, Brain Tumor Center at ErasmusMC Cancer Institute, Rotterdam, The Netherlands; 28 Institute for Experimental Pathology, ARUP Laboratories, Salt Lake City, UT USA; 29 Department of Pathology, University of Utah and ARUP Laboratories, Salt Lake City, UT USA; 30 Department of Neurosurgery, Vivantes Klinikum im Friedrichshain, Berlin, Germany; 31 Department for Pathology, Katharinenhospital Stuttgart, 70174 Stuttgart, Germany; 32 Department of Neurosurgery, Heidelberg University Hospital, Heidelberg, Germany; 33 Department of Neuropathology and Center for Behavioral Brain Sciences (CBBS), Otto-von-Guericke-University Magdeburg, and Center of Behavioral Brain Science, Magdeburg, Germany; 34 Centre for Regenerative Medicine, Institute for Regeneration and Repair, University of Edinburgh, Edinburgh, UK; 35 Department of Pathology, University Medical Center Utrecht, Utrecht, The Netherlands; 36 Department of Neurosurgery, School of Medicine, Acibadem University, 34752 Istanbul, Turkey; 37 Division of Pediatric Neurooncology, German Cancer Consortium (DKTK), German Cancer Research Center (DKFZ), Heidelberg, Germany; 38 Department of Pediatric Hematology and Oncology, Heidelberg University Hospital, Heidelberg, Germany; 39 Pathology Unit, Department of Molecular and Translational Medicine, University of Brescia Medical School, Brescia, Italy; 40 Signalling Research Centres BIOSS and CIBSS, University of Freiburg, Freiburg, Germany; 41 Institute of Neuropathology, Faculty of Medicine, University of Freiburg, Freiburg, Germany; 42 Department of Neuropathology, Regensburg University Hospital, Regensburg, Germany; 43 NYU Langone Health, New York, NY USA; 44 Princess Margaret Cancer Centre, MacFeeters Hamilton Neuro-Oncology Program, University Health Network and University of Toronto, Toronto, ON Canada; 45 Division of Neurosurgery, Department of Surgery, University of Toronto, Toronto, ON Canada; 46 Department of Pathology, Amsterdam University Medical Centers, Amsterdam and Princess Máxima Center for Pediatric Oncology, Utrecht, The Netherlands; 47 Clinical Cooperation Unit Neurooncology, German Consortium for Translational Cancer Research (DKTK), German Cancer Research Center (DKFZ), Heidelberg, Germany

**Keywords:** Astrocytoma, IDH-mutant, Primitive neuronal component, DNA methylation, *RB1*, *MYCN*

## Abstract

IDH-mutant astrocytomas are diffuse gliomas that are defined by characteristic mutations in *IDH1* or *IDH2* and do not have complete 1p/19q co-deletion. The established grading criteria include histological features of brisk mitotic activity (grade 3) and necrosis and/or microvascular proliferation (grade 4). In addition, homozygous deletion of the *CDKN2A/B* locus has recently been implemented as a molecular marker for grade 4 IDH-mutant astrocytomas. Here, we describe a subgroup of high-grade *IDH*-mutant astrocytomas characterised by a primitive neuronal component based on histology and a distinct DNA methylation profile (n = 51, ASTRO PNC). Misinterpretation as carcinoma metastasis was common, since GFAP expression was absent in the primitive neuronal component, whereas TTF-1 expression was detected in 15/19 cases (79%) based on immunohistochemistry. Apart from mutations in *IDH1*, *TP53*, and *ATRX*, we observed enrichment for alterations in *RB1* (n = 19/51, 37%) and *MYCN* (n = 14/51, 27%). Homozygous *CDKN2A/B* deletion (n = 1/51, 2%) and *CDK4* amplification (n = 3/51, 6%) were relatively rare events. Clinical (n = 31 patients) and survival data (n = 23 patients) indicate a clinical behaviour similar to other CNS WHO grade 4 IDH-mutant astrocytomas, however with an increased risk for leptomeningeal (n = 7) and extra-axial (n = 2) spread. Taken together, ASTRO PNC is defined by a distinct molecular and histological appearance that can mimic metastatic disease and typically follows an aggressive clinical course.

**Supplementary Information:**

The online version contains supplementary material available at 10.1007/s00401-025-02849-8.

## Introduction

Isocitrate dehydrogenase (IDH)-mutant astrocytomas are diffuse gliomas with a defining mutation in *IDH1* or *IDH2* without complete 1p/19q co-deletion. Mutations in *alpha thalassemia/mental retardation syndrome, X-linked* (*ATRX*), and *tumour protein 53* (*TP53)* are further defining molecular features. This is different from another distinct entity of *IDH*-mutant gliomas being the oligodendrogliomas, IDH-mutant with complete 1p/19q co-deletion. In oligodendroglioma, *telomerase reverse transcriptase* (*TERT*) promoter mutations are characteristic and *capicua transcriptional repressor* (*CIC*) and *Far Upstream Element [FUSE] Binding Protein 1* (*FUBP1*) alterations are also enriched. The established grading criteria for IDH-mutant astrocytomas are histological criteria including brisk mitotic activity (CNS WHO grade 3), as well as necrosis and/or microvascular proliferation (CNS WHO grade 4). Recently, homozygous deletion of the *cyclin-dependent kinase inhibitor 2A/B* (*CDKN2A/B*) locus was added as grading criterium for CNS WHO grade 4 IDH-mutant astrocytomas [4, 8, 13]

Since 2020, novel subgroups of IDH-mutant gliomas defined by unique DNA methylation profiles have been reported [5]. These subgroups can be additionally linked to characteristic morphological appearances like sarcomatous features in oligosarcomas [16], mutation profiles such as hypermutation and mismatch-repair-deficiency in primary mismatch-repair-deficient IDH‐mutant astrocytomas (PMMRDIAs) [17] or special localisations as for infratentorial IDH-mutant astrocytomas [2].

In 2021, we have described a subgroup of IDH-wildtype glioblastomas that harbour a primitive neuronal component (GBM PNC) and a distinct DNA methylation profile. These tumours frequently show expression of TTF-1 (a transcription factor most often detected in tissues of pulmonary or thyroidal origin), absence of GFAP expression and propensity for leptomeningeal spread [9, 18]. A few cases of similar IDH-mutant astrocytomas with a primitive component have been described in literature [6, 10, 14]. Here, we describe the mutational and epigenetic profile of IDH-mutant astrocytomas with primitive neuronal component (ASTRO PNC), characterised by similar histological features as well as a dismal clinical outcome.

## Materials and methods

### Collection of tissue samples and clinical data

Identification of this series was achieved by unsupervised visualisation of genome-wide DNA methylation data collected of uploaded samples to the http://www.molecularneuropathology.org platform. This visualisation revealed a distinct subset of cases. This subset was validated in a supervised visualisation in a tSNE analysis with selected IDH-mutant brain tumours and IDH-wildtype glioblastomas with primitive neuronal component (20,000 most variable CpG methylation sites, 3000 iterations, perplexity value of 20) with the R package rtsne. The chosen reference samples were from the University Hospital Heidelberg (thereafter Heidelberg) with highest scores in the brain tumour classifier for those entities and from previously published series.

### Methylation and summary CNV plot

For DNA methylation, the Infinium HumanMethylation450 (450 k) BeadChip or Infinium MethylationEPIC (850 k) or EPICv2.0 BeadChip array (Illumina, San Diego, USA) was used according to the manufacturer’s instructions. The data were processed as previously described [3]. Summarisation of the copy number variations was generated by dividing the chromosomal arms into 1% segmental bins and plotting the frequency of gains and losses using the ggplot2 R package.

### Histology and immunohistochemistry

Histology and immunohistochemistry were performed centrally in Heidelberg when sufficient tumour material was available (n = 21). Immunohistochemical staining was performed on a Ventana BenchMark ULTRA Immunostainer (Ventana Medical Systems, Tucson, USA) on formalin-fixed and paraffin-embedded (FFPE) sections. The immunohistochemical panel covered staining for GFAP (clone GA5, dilution 1:2000, Cell Signaling, Danvers, MA, USA), IDH1 R132H (in house, clone 14/10, dilution 1:2), TTF-1 (clone EP229, dilution 1:50, Cell Marque, Rocklin, USA), NSE (clone MIG-N3, dilution 1:4, Linaris, Biozol, Eching, Germany), Chromogranin A (clone LK2H10, RTU, Linaris, Biozol, Eching, Germany), Neu-N (clone A60, dilution 1:100, Merck, Darmstadt, Germany), Olig2 (clone EPR2673, dilution 1:50, Abcam), Ki67 (clone MIB-1, dilution 1:100, Dako Agilent, Santa Clara, CA, USA), synaptophysin (clone MRQ-40, dilution 1:50, Merck), ATRX (clone BSB-108, dilution 1:2000, Bio SB, Santa Barbara, CA, USA), cytokeratins (clone AE1 & clone AE3, RTU, DCS Hamburg, Germany) and p53 (clone DO-7, dilution 1:50, Novocastra, Leica, Wetzlar, Germany). Stained slides were scanned on the Aperio AT2 Scanner (Aperio Technologies, Vista, USA) and digitalized using Aperio ImageScope software v12.3.2.8013.

### Targeted DNA and RNA sequencing

For 14 cases sequenced in Heidelberg, DNA was enriched with Agilent SureSelect technology on a Panel of 201 tumour-relevant genes as described before [12]. Sequencing was performed on Illumina Novaseq6000 according to the manufacturer’s instructions. The dataset was filtered for exonic regions and selected intronic regions. Exonic and splicing indels and nonsynonymous single-nucleotide variants (SNVs) with a frequency of ≤ 0.001 in the 1000 genomes database (https://www.internationalgenome.org/) were identified after subtracting low-quality calls. For four other cases, a local targeted panel containing all relevant genes for brain tumour diagnostics was used. For two cases, whole-exome sequencing was already performed.

For six cases, RNA sequencing was performed as described previously [15]. Fusion calling was done using the Arriba algorithm [19].

### Statistics

Kaplan–Meier estimates, log-rank tests, their corresponding figures and pie charts were performed using GraphPad Prism (GraphPad Inc., Boston, USA). For survival data, day 0 was set to the date of surgery (for ASTRO PNC). For Fig. 6a, an image from Servier Medical Art (http://smart.servier.com/) was altered licenced under a Creative Commons Attribution 3.0 Unported 0.. Sample sizes are indicated with *n*.

## Results

### IDH-mutant astrocytomas with a primitive neuronal component (ASTRO PNC) share a distinct DNA methylation profile

**Fig. 1:**
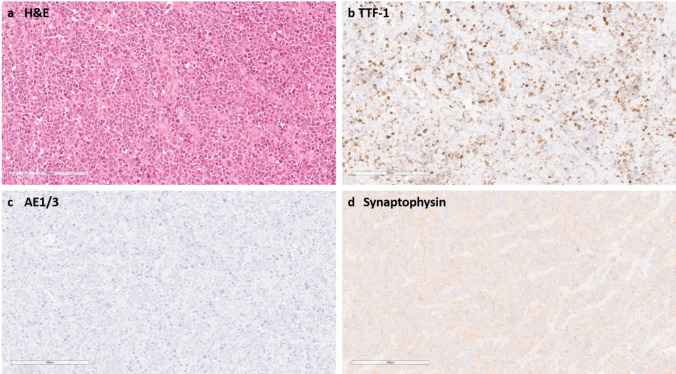
**a** The index case presents itself with a small blue round cell morphology with mitotic figures and occasional cell wrapping. **b** TTF-1 immunohistochemistry is expressed in the tumour cells. **c** High-molecular-weight cytokeratins were not expressed. **d** Synaptophysin is expressed. Scale bar denotes 200 µm

**Fig. 2:**
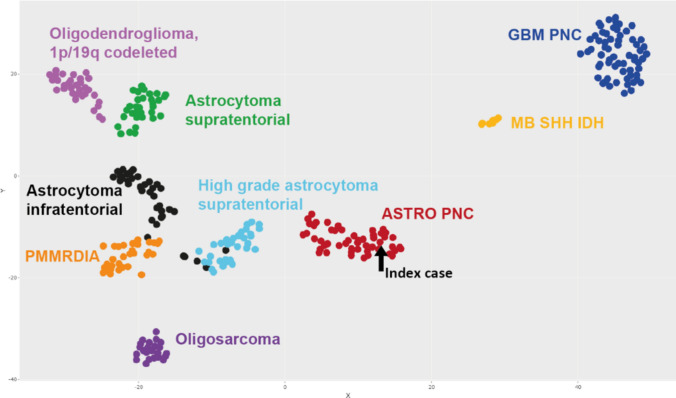
Methylation data: in a tSNE analysis with selected reference entities from our database (n = 310), astrocytoma, IDH-mutant, PNC separates clearly from the other entities. PMMRDIA, primary mismatch-repair-deficient IDH-mutant astrocytoma; MB SHH IDH, medulloblastoma, SHH-activated, IDH-mutant; GBM PNC, glioblastoma, IDH-wildtype, with primitive neuronal component. The methylation class astrocytoma, IDH-mutant, high grade consists of 28 grade 4 tumours and 3 grade 3 tumours. The methylation class astrocytoma, IDH-mutant, low grade consists of 2 grade 4 tumours, 13 grade 3 tumours and 17 grade 2 tumours

During routine diagnostics of brain tumours, we encountered an index case featuring “small blue round-cell” morphology, expression of TTF-1 and diffuse expression of synaptophysin. Histologically, the case resembled a small cell carcinoma metastasis. However, high-molecular-weight cytokeratin staining was negative (Fig. 1). After molecular analyses and further immunohistochemical staining, an IDH1 R132H mutation and a DNA methylation class prediction of “(high-grade) astrocytoma IDH-mutant” (in Heidelberg classifier v12.5) were obtained. Sampling of additional material identified additional areas of a diffusely infiltrating glioma consistent with an astrocytoma. We subsequently clustered the case with 176,857 DNA methylation profiles of tumour samples and control brain tissue samples (including local and external cases submitted through molecularneuropathology.org) and found a larger group of cases that were clustering in close relationship to the initial sample, most often with the highest score for IDH-mutant astrocytoma. For multiple cases, the referred diagnoses suggested a primitive neuroectodermal tumour or suspicion for metastasis. In total, we identified 51 samples with such a distinct methylation profile (Fig. 2).

After re-analysing the identified samples using the latest (v12.8) version of the Heidelberg brain tumour classifier, most samples (n = 39/51, 76.5%, Online Resource in supplementary Table 1) were successfully allocated to the methylation class of astrocytoma, IDH-mutant; high grade (calibrated score > 0.9). Because of the presence of a primitive neuronal component in all of these tumours, we named the distinct methylation group “astrocytoma, IDH-mutant, with primitive neuronal component” (ASTRO PNC). This methylation group was clearly distinct from the earlier described methylation classes of glioblastomas with primitive neuronal component (GBM PNC) and high-grade IDH-mutant astrocytomas, that include grade 4 IDH-mutant astrocytomas [(n = 28/31), Fig. 2].

### ASTRO PNCs express TTF-1

**Fig. 3:**
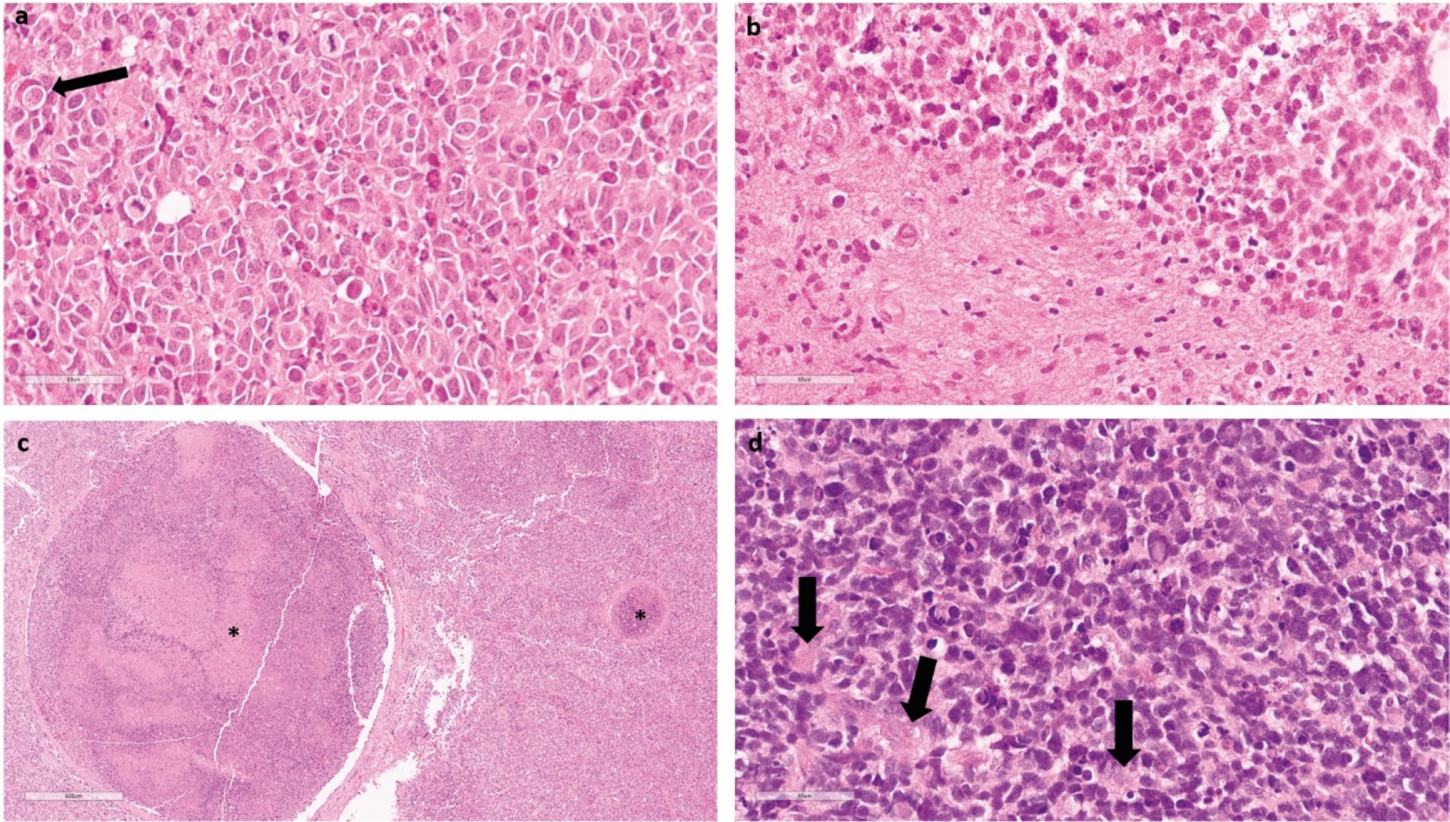
**a** All cases investigated by histology harbour a primitive neuronal component with small blue round tumour cells. In addition, in this case, cell wrapping (arrow) can be observed. **b** In most cases, a sharp demarcation of the primitive neuronal component and the astrocytic component can be found. **c** Presence of tumour growth inside the lumina of two vessels (asterisks). Despite reported extra-axial spread in this tumour entity, this case does not have reported metastases: **d** some cases present themselves with rosettes (arrows). Scale bar denotes 60 µm in **a**, **b** and **d**. Scale bar denotes 600 µm in **c**

**Fig. 4:**
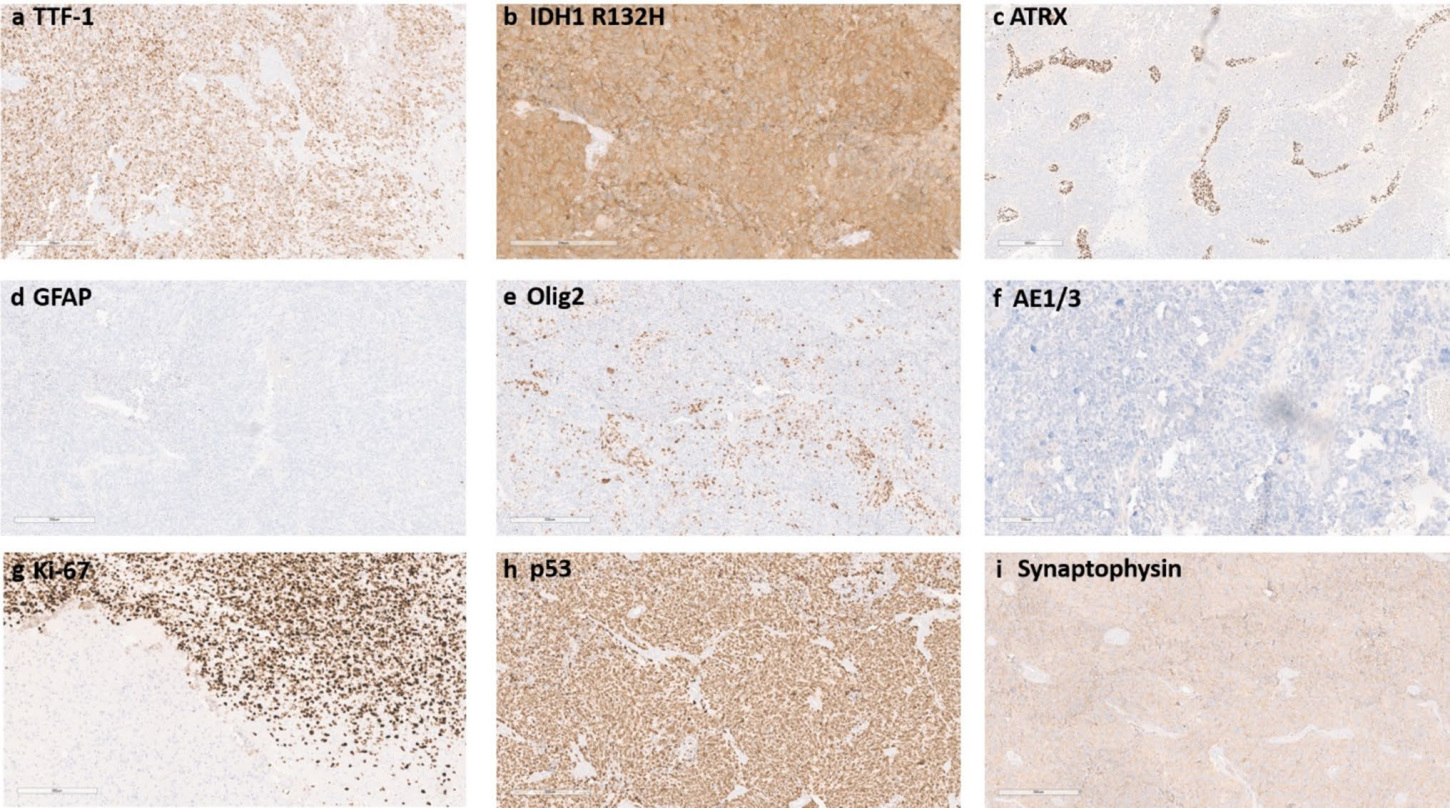
**a** TTF-1: the primitive neuronal component expresses TTF-1 (using TTF-1 clone EP229). In a subset of cases, the expression is limited to a small number of cells. **b** IDH1 R132H: most tumour cases strongly express IDH1 R132H. **c** ATRX: nuclear ATRX expression is lost in most of the cases with blood vessels suited as positive internal controls. **d** GFAP: GFAP expression is lost in the primitive neuronal component. **e** Olig2: a subset of the primitive tumour cells expresses Olig2. **f** AE1/3: the tumours do not express high-molecular-weight cytokeratins stained with AE1 and/or AE3. **g** Ki-67: the proliferation index is high in the primitive neuronal component, whilst in most cases, the glial component does not show an elevated proliferation index. **h** p53: p53 is strongly expressed in the primitive tumour cells. **i** Synaptophysin: synaptophysin is expressed in the primitive tumour cells. Scale bar denotes 300 µm

To assess the histology of the new methylation group, we collected tumour samples from 21 patients. All tumour samples (partially) presented with areas of astrocytic differentiation with a fibrillary matrix formed by astrocytic cell processes, a pattern that is usually observed in IDH-mutant astrocytomas. The round to oval nuclei showed nuclear atypia regarding chromatin density and size (Fig. 3). Using immunohistochemical staining, expression of mutant IDH1 (R132H) was detected in the majority of cases (n = 18/20, 90%) and a loss of ATRX expression (n = 15/16, 94%) was equally common. The p53 protein was accumulated in most observed cases (n = 14/15, 93%) (Fig. 4). For the two cases lacking IDH1 R132H expression, different IDH1 R132 substitutions were detected in the sequencing analysis (IDH1 R132S in patient 10 and IDH1 R132S in patient 3; Online Resource in supplementary Table 1).

In contrast to the vast majority of IDH-mutant astrocytomas, all cases also showed a primitive neuronal component with strong nuclear expression of TTF-1, diffuse synaptophysin positivity and in a subset of cases positivity for NSE and Neu-N (Online Resource in supplementary Table 1). The morphology of the primitive neuronal component was heterogeneous with rosettes, cell wrapping and a small-blue-round-cells (Fig. 3). In addition, a high proliferation index above 85% was generally observed in the primitive area of the tumour. The PNC showed clear differences regarding the Ki-67, TTF-1 and GFAP expressions levels compared to the astrocytic component (Fig. 4). Similar to the astrocytic component, IDH1 R132H was expressed and p53 was accumulated, whilst ATRX expression was lost in the primitive component. In line with the expected glial origin, Olig2 was expressed in a subset of primitive cells as well. To check for TTF-1 cross-reactivity, we stained several non-neoplastic tissue and other samples to investigate TTF-1 expression in non-neoplastic CNS tissue and IDH-mutant astrocytomas. In line with our expectations, TTF-1 was not expressed in non-neoplastic CNS tissue and only 3.5% (1/28) of high-grade astrocytoma CNS WHO grade 4, with one case showing single tumour cells being positive

Necrosis was present in n = 17/20 (85%) cases. Microvascular proliferation was present in 18/20 (90%) cases. Multiple mitoses could be observed in 19/20 (95%) cases. Thus, based on the WHO CNS5 grading criteria, all tumours presented with histological criteria warranting an IDH-mutant astrocytoma CNS WHO grade 4 designation due to either presence of necrosis or vascular proliferation (Online Resource in supplementary Table 1).

### ASTROs PNC show recurrent mutations in *RB1*

**Fig. 5:**
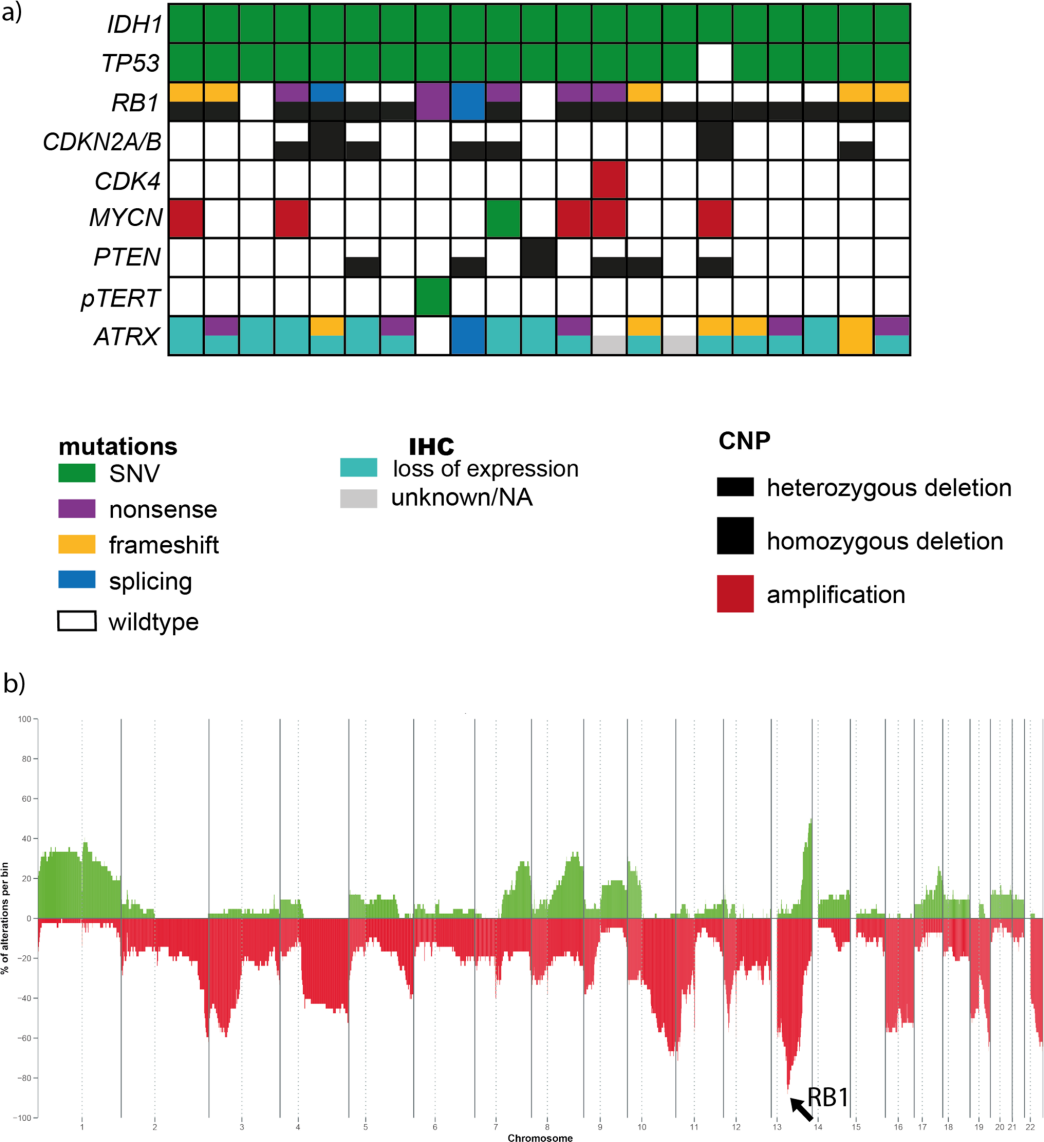
**a** Oncoprint of 21 samples for which DNA targeted panel sequencing was performed, including genetic mutations, immunohistochemistry (IHC) and copy number variations calculated from the DNA methylation array. **b** The copy number summary plot (n = 42) reveals a focal deletion at the RB1 locus in addition to several chromosomal gains and losses

To evaluate the mutational profile of our cohort, we performed next-generation sequencing with a custom Illumina Panel on 21 tumour samples. Besides the characteristic *IDH1* mutation (n = 21/21, 100%, thereof n = 19/21, 90% IDH1 R132H mutant), alterations were detected in *TP53* (n = 20/21, 95%) and in the telomere maintenance mechanism (TMM) including *ATRX* (n = 18/21, 86%) and the *TERT* promoter (*pTERT)* (n = 1/21, 5%). For IDH-mutant astrocytomas without a primitive neuronal component, the usual TMM alteration is a loss of function event on the *ATRX* gene. In addition, we observed mutations in *RB1* in 12/21 cases (57%, Fig. 5a).

### ASTROs PNC show recurrent copy number alterations in *RB1* and *MYCN*

Copy number profiles based on DNA methylation array analysis were performed on all 51 samples and frequently showed deletion of chromosome arm 13q with deletions of *RB1* (homozygous deletions in n = 5/51, 10%; hemizygous deletions in n = 38/51, 75%). A gain of chromosome 1 and a loss of chromosome arms 3p, 4q, 16, 19 and 22q were commonly observed as well (Fig. 5b). *MYCN* was frequently altered in ASTRO PNCs, with a subset of cases showing *MYCN* amplifications in the copy number variation profile. One case harboured a pathogenic *MYCN* mutation (*MYCN* alterations n = 14/51, 27%). RNA sequencing performed on six samples did not show any relevant fusion events.

In contrast to GBM PNC, the *PTEN* locus was mostly balanced in ASTRO PNC, with only two cases showing a homozygous *PTEN* deletion (n = 2/51, 4%). *PTEN* deletions are rarely observed in IDH-mutant astrocytomas. High-grade IDH-mutant astrocytomas often show cell cycle deregulation due to homozygous *CDKN2A/B* deletions or amplifications of *CDK4* or *CDK6*. However, these were only rare events in ASTRO PNCs with only a single case showing homozygous *CDKN2A/B* deletion (n = 1/51, 2%) and three cases with *CDK4* amplification (n = 3/51, 6%). *CDK6* amplifications were not detected in our cohort.

### Tumour location shifts from the frontal to the temporal lobe during progression

**Fig. 6:**
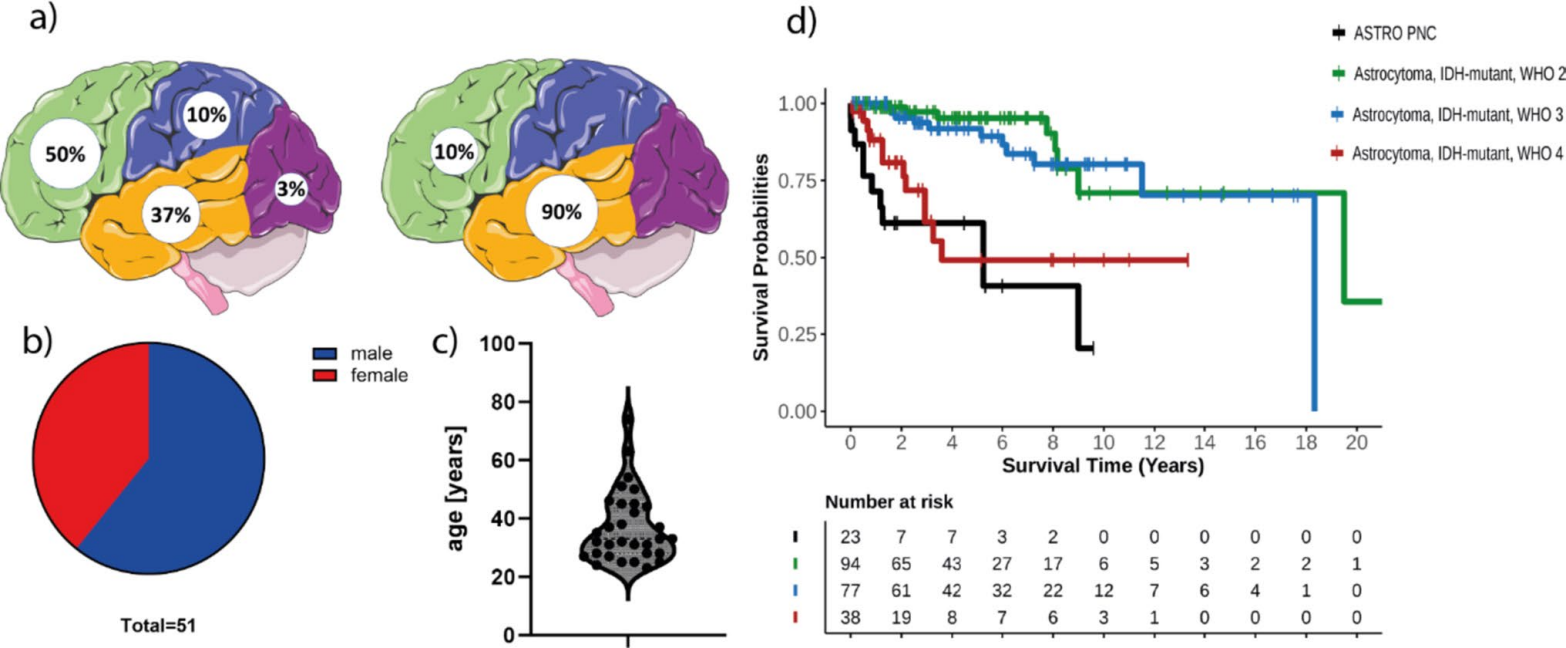
**a** Left side: the most common site of origin for primary diagnoses of ASTRO PNC is the frontal lobe followed by the temporal lobe. Right side: for recurrent ASTRO PNC cases, the most common site is the temporal lobe. **b** The tumours show a predisposition for the male sex. **c** Age distribution in ASTRO PNC (n = 30, median age = 38). **d** Kaplan–Meier estimate indicates no significant difference to IDH-mutant astrocytomas WHO grade 4 (*p* = 0.24). In contrast, there is a significant difference to IDH-mutant astrocytomas WHO grade 2 and 3 (*p*(A2) = < 0.0001; *p*(A3) = < 0.0001)

We also investigated whether, in addition to a distinct DNA methylation profile, ASTRO PNCs are associated with different clinical features compared to IDH-mutant astrocytomas without a primitive neuronal component. Therefore, we collected clinical data for 31 patients with ASTRO PNCs and compared them to clinical data from 38 patients with IDH-mutant astrocytomas CNS WHO grade 4 without a primitive neuronal component. Whilst IDH-mutant gliomas are predominantly located in the frontal lobe, ASTRO PNCs were more often located in the temporal lobe as well as the frontal lobe (Fig. 6a). For primary lesions (n = 15, 50%), the frontal lobe was the preferred location (Fig. 6a, left side), whilst recurrent tumours (n = 10, 90%) were enriched in the temporal lobe (Fig. 6a, right side), similar to our observations in IDH-wildtype glioblastomas with primitive neuronal components (52% located in the temporal lobe [18]). As we did not have any matched pairs of primary and recurrent tumours in our cohort, we do not know if primary lesions of recurrent tumours were also described to have a primitive neuronal component. We did not observe any frontal-to-temporal lobe transition during recurrence in a reference cohort of IDH-mutant gliomas (including 56 non-PNC astrocytomas and 48 oligodendrogliomas).

### Sex, age and *MGMT* promoter methylation similar to CNS WHO grade 4 IDH-mutant astrocytomas

For further characterisation, we compared median age and sex distribution of patients with ASTRO PNCs to those of patients with IDH-mutant astrocytomas CNS WHO grade 4 without a primitive neuronal component. Similar to IDH-mutant astrocytomas, we observed a male predominance (61%, Fig. 6b). Median age at diagnosis of an ASTRO PNC was 38 years compared to 42 years in an internal reference cohort of 38 patients with IDH-mutant astrocytomas CNS WHO grade 4 without a primitive neuronal component (p = 0.08, [11], Fig. 6c). The *MGMT* promoter was methylated in 90% of ASTRO PNCs (n = 46/51).

Of note, looking at methylation of all CpGs included on the methylation array, we observed a high rate of unmethylated CpG probes in ASTRO PNCs, which may indicate a more aggressive clinical course in IDH-mutant astrocytomas (G-CIMP low), comparable to IDH-wildtype glioblastomas and high-grade IDH-mutant astrocytomas (Supp. Figure 1).

### Frequent leptomeningeal dissemination and poor survival similar to patients with CNS WHO grade 4 IDH-mutant astrocytomas

To examine the prognostic relevance of these tumours, we evaluated survival data for 23 patients. We performed a Kaplan–Meier estimate and compared our cohort to an internal cohort of 209 IDH-mutant astrocytomas of CNS WHO grades 2, 3, or 4 (Fig. 6d). The overall survival of patients with ASTRO PNC and IDH-mutant astrocytoma CNS WHO grade 4 patients showed no significant difference (p = 0.2446) but were significantly different to patients with IDH-mutant astrocytomas of CNS WHO grades 2 or 3 (p(A2) = < 0.0001; p(A3) = < 0.0001). Furthermore, IDH-mutant astrocytomas with primitive neuronal component showed a higher risk of leptomeningeal spread with seven patients developing leptomeningeal and/or subarachnoidal dissemination intracranially and in the spine during progression. Two patients had metastases outside of the central nervous system (Table 1).

**Table 1:** Clinical data of the patients of the core cohort

case ID	PFS (moment)	OS (months)	Alive (0 = yes, 1 = no)	Localisation (lobe)	Age (years)	Site of extra-axial manifestation	Leptomeningeal spread
1	0	6	1	Frontotemporal	33	Subcutaneous lymph node	Dural
2	3	NA	0	Temporal	38	0	0
3	NA	NA	NA	NA	NA	NA	NA
4	0.16	0.16	1	Temporal	51	0	0
5	15	15	1	Frontal	33	NA	NA
6	38	63	1	Temporal	46	0	0
7	24	63	1	Temporal	28	0	T2 to T4
8	16	16	0	Parietal	23	NA	NA
9	22	22	0	Frontal	74	NA	NA
10	44	64	0	Temporal	25	NA	NA
11	36	54	0	Temporal	32	NA	NA
12	63	115	0	Parieto-occipital	50	0	0
13	NA	NA	NA	Frontal	45	0	0
14	22	22	0	Temporal	31	0	0
15	NA	72	0	Frontal	63	NA	NA
16	NA	1.50	0	Frontal	42	0	0
17	87	108	1	Frontal	32	NA	NA
18	2	2	1	Temporal	25	NA	Ventricles, C7 to B8; later until cauda equina
19	NA	72	0	Frontal	28	NA	NA
20	NA	NA	NA	Frontal	44	NA	NA
21	NA	16	0	Temporal	31	NA	NA
22	0	0	1	Temporal	35	0	1
23	2.99	2.99	0	Temporal	31	NA	NA
24	NA	10.06	1	Temporal	45	NA	NA
25	8	14	1	Frontal	27	liver, bone	1
26	NA	6	1	Frontal	28	0	0
27	NA	NA	NA	Temporal	37	0	0
28	NA	NA	NA	Frontal	27	0	0
29	NA	NA	NA	Temporal	24	0	0
30	NA	NA	0	Temporal	25	NA	Cervical, thoracic
31	12	20.75	0	Temporal	54	0	1

Table 1 Progression-free survival (PFS), overall survival (OS) both rounded, survival status, localisation, age, extra-axial manifestations and leptomeningeal spread.

## Discussion

Here, we present a new molecular subtype of astrocytoma, IDH-mutant that shows a characteristic histological pattern paired with a distinct epigenetic profile. The primitive component lacks a particular highly differentiated growth pattern and GFAP expression, but expresses TTF-1, displays features of neuronal differentiation as evidenced by diffuse synaptophysin positivity and shows a high proliferation index. These characteristics can raise the differential diagnosis of a metastatic small cell carcinoma; however, without cytokeratin expression. Moreover, these tumours often contain additional areas of astrocytic differentiation and display a typical immunohistochemical profile of IDH-mutant astrocytoma, with loss of nuclear ATRX expression, nuclear accumulation of p53, and positive staining for IDH R132H in the vast majority of tumours. Furthermore, most ASTROs PNC harbour a methylated MGMT promoter, which is a molecular feature similar to conventional IDH-mutant astrocytomas. In contrast to the usual frontal localisation of IDH-mutant astrocytomas, these tumours are predominantly found in the temporal lobe. In addition, leptomeningeal/subarachnoidal and/or extra-axial dissemination is often observed (Table 1). The clinical outcome is comparable to that of other patients with IDH-mutant astrocytoma, CNS WHO grade 4. Based on our findings, we suggest calling this subgroup “astrocytoma, IDH-mutant, with primitive neuronal component (ASTRO PNC)”.

We did observe TTF-1 expression with the TTF-1 EP229 clone in ASTRO PNCs. From our previous studies, a negative staining with this clone was shown for glioblastomas without a primitive neuronal component [18]. It is well known that different TTF-1 clones show different reactivity in glioblastomas and non-neoplastic CNS tissue [7, 10, 18]. We did find faint TTF-1 expression in a single case of astrocytoma, IDH-mutant, CNS WHO grade 4 without primitive component, indicating a high sensitivity but not specificity for the TTF-1 EP229 clone for primitive neuronal component, or perhaps the detection of a tumour that is in the process of transitioning to ASTRO PNC. In addition, TTF-1 is known to be expressed during foetal brain development, especially in the ventral forebrain [3, 7]. This might indicate that the tumours have gone a pan-cancer convergence to a small-cell phenotype similar to as it was described by Balanis et al. for epithelial tumours with acquired *TP53* and *RB1* mutations [1].

In recent years, molecular markers have entered routine neuropathological diagnostics for typing and grading of CNS tumours. With the fifth edition of the WHO classification of Tumours of the Central Nervous System (CNS), presence of homozygous CDKN2A/B deletions mandates a CNS WHO grade 4 designation of IDH-mutant astrocytoma independent of histological features. According to a recent study by Weller et al., 17.1% of CNS WHO grade 4 IDH-mutant astrocytomas carry a homozygous CDKN2A/B deletion [20]. In ASTROs PNC, only one tumour showed a homozygous CDKN2A/B deletion, whilst *MYCN* amplifications and *RB1* alterations were frequently observed. These alterations have been discussed as potential adverse prognostic markers, but they are currently not implemented as molecular grading criteria [4]. The loss of function alterations in *RB1* can result in a more downstream deregulation of cell cycle arrest, in fact in the same pathway as affected by *CDKN2A/B* deletions, as previously described [18]. In addition, mutations in *TP53,* whose gene product is involved in cell cycle arrest and regulation of DNA damage repair*,* and *RB1* alterations do co-occur in most of the sequenced ASTRO PNCs (n = 12/21, 57%).

Similar to glioblastomas with primitive neuronal component, we observed a higher frequency of localisation in the temporal lobe, which is even more pronounced in recurrent tumours, and thus present further similarities between IDH-wildtype and IDH-mutant astrocytic gliomas with a primitive neuronal component. For recurrent tumours, we do not know whether the corresponding primary tumours already showed a primitive neuronal component histology and originated in the same location. However, the finding that 10 of 25 cases with clinical annotation were recurrent tumours indicates that ASTRO PNC might represent progression of IDH-mutant astrocytomas towards a more malignant phenotype, similar to IDH-mutant oligodendrogliomas progressing to oligosarcomas [16].

The risk of leptomeningeal spread and extra-axial metastases, which are rare clinical events in IDH-mutant astrocytoma patients in general, appears to be increased in patients with ASTRO PNCs. The results indicate the need for close radiological follow-up and heighted awareness for somatic symptoms in patients diagnosed with an ASTRO PNC. However, due to the comparably small cohort and limited clinical data for a substantial fraction of patients in our cohort, a follow-up study is needed to confirm these observations.

In conclusion, our findings suggest that ASTROs PNC can be regarded as a distinct subtype of IDH-mutant astrocytomas. The clinical course of ASTRO PNCs corresponds to astrocytoma, IDH-mutant, CNS WHO grade 4, and patients should be monitored for leptomeningeal and extra-axial spread. The high scores obtained for the methylation class “astrocytoma, IDH-mutant, high-grade” in the Heidelberg brain tumour classifier v12.8 indicate the profound impact of IDH-mutation on global DNA methylation. Future versions of the brain tumour classifier should recognise this subtype as a distinct methylation class, similar to the integration of the methylation class of oligosarcomas into more recent versions of the CNS tumour classifier. Furthermore, we propose testing for TTF-1 and neuronal marker expression in IDH-mutant gliomas showing primitive features to filter out patients that might benefit from extensive clinical monitoring. Conversely, when an intracerebral tumour is detected with small cell phenotype, TTF-1 positivity and absence of GFAP staining in the tumour cells, additional testing for high-molecular-weight cytokeratin, Olig2 expression, mutant IDH1 (R132H) and potentially also IDH-sequencing can prevent identifying the lesion incorrectly as a metastasis instead of ASTRO PNC.

## Supplementary Information

Below is the link to the electronic supplementary material.

Supplementary file1 (XLSX 22 KB)

## Data Availability

Upon reasonable request, the data presented in this manuscript including DNA methylation, mutational, and clinical outcome data can be shared.
